# LIPUS as a potential strategy for anti-inflammation and repair: A review of the mechanisms

**DOI:** 10.7150/ijms.124996

**Published:** 2026-02-18

**Authors:** Gaocheng Wang, Jianping Zhao, Jiaxuan Li, Zhanguo Zhang, Wanguang Zhang, Qian Chen, Jingjing Wang

**Affiliations:** 1 Hepatic Surgery Center, Tongji Hospital of Tongji Medical College of Huazhong University of Science and Technology, Wuhan, Hubei, China; Hubei Key Laboratory of Hepato-Pancreato-Biliary Diseases, Wuhan, China.; 2 The Second Clinical Department, Tongji Medical College, Huazhong University of Science and Technology, Wuhan, China.; 3 Division of Gastroenterology, Department of Internal Medicine at Tongji Hospital, Tongji Medical College, Huazhong University of Science and Technology, Wuhan, China.; 4 Department of Medical Ultrasound, Tongji Hospital, Tongji Medical College, Huazhong University of Science and Technology, Wuhan, China.

**Keywords:** low-intensity pulsed ultrasound (LIPUS), inflammation, repair, signaling pathway, molecular mechanism

## Abstract

Hundreds of millions of people worldwide endure continuous suffering and significant economic burdens due to inflammatory diseases. Various acute and chronic inflammatory diseases and the natural aging of the human body are common causes of organ damage. Therefore, how to reasonably regulate inflammation, tissue repair and regeneration after organ damage has been of great concern, especially the pathological repair caused by inflammation will lead to the destruction of the original structure and function of tissues and organs. Low-intensity pulsed ultrasound (LIPUS) is a promising non-invasive physical therapy that can produce different biological effects on organs, tissues and cells. Certain clinical trials have demonstrated the outstanding capacity of LIPUS in anti-inflammation and repair. Many* in vivo* and *in vitro* basic studies have also reported the molecular effect mechanisms by which LIPUS exerts capacity of anti-inflammation and repair. This review focuses on the molecular mechanism of LIPUS anti-inflammation and repair and emphasizes the crucial role of LIPUS in various diseases. In addition, we compile clinical trials to provide readers with a more thorough understanding of the current potential of LIPUS in inflammation control and organ function restoration.

## 1. Introduction

The global burden of immune-mediated inflammatory diseases is severe. In 2019, approximately 67.58 million people were affected worldwide, leading to significant health impairments and socioeconomic consequences 1. Chronic inflammatory diseases are recognized as the leading cause of death globally, with more than 50% of deaths attributed to inflammation-related disorders 2. A variety of acute and chronic inflammatory diseases and the natural aging of the human body are common drivers of organ damage. Therefore, the rational regulation of inflammation, tissue repair, and regeneration after organ damage has always been a significant concern. In particular, the pathological repair induced by inflammation can destroy the original structure and function of tissues and organs, such as skin keloid, emphysema and pulmonary fibrosis, cirrhosis of the liver, glomerulosclerosis, or cerebral gliosis 3.

Current treatments for inflammatory diseases include antibiotics, steroids, and immunosuppressive drugs, which are usually administered systemically 4. Despite advances in existing targeted therapies, patient remission rates remain suboptimal, and achieving sustained remission without medication remains an unmet clinical need 1. For instance, in 2021, over 22% of adults over 40 had knee osteoarthritis, with end-stage patients often requiring joint replacement. Currently, no cure exits for osteoarthritis. Clinical treatments traditionally focus on symptom relief rather than halting disease progression 5. Consequently, there is an urgent need to develop novel therapeutic strategies.

Low intensity pulsed ultrasound (LIPUS) is widely regarded as a non-invasive physical therapy. Due to its relatively low intensity, the thermal effects of LIPUS are negligible 6. An increasing body of evidence demonstrates that LIPUS possesses potent anti-inflammatory properties and significantly promotes the structural and functional repair of tissues and organs 7-10. Given its therapeutic efficacy, targeted approach, non-invasiveness, and convenience, LIPUS represents an ideal modality for anti-inflammation and repair. Recent studies suggest that LIPUS holds significant promise for both anti-inflammatory and tissue protection. For instance, Li *et al.* 11 highlighted the major molecular mechanisms through which LIPUS exerts anti-inflammatory effects.

Key molecules and cell process are involved in both the inflammatory response and the protection and restoration of organ structure and function. These include focal adhesion kinase (FAK) 12, brain-derived neurotrophic factor (BDNF) 13 and transforming growth factor-β (TGF-β) 14. Furthermore, LIPUS regulates many of these shared key molecules. However, comprehensive reviews addressing this dual role are lacking. Therefore, this review summarizes the molecular mechanisms by which LIPUS therapy facilitates both anti-inflammation and tissue repair, exploring its potential in treating various diseases. LIPUS has been shown to inhibit inflammation and fibrosis in conditions such as chronic prostatitis 15, osteoarthritis 16, cardiovascular diseases 10, 17, and renal injuries 18. In neuro-related disorders, it supports nerve repair 19, and it also aids in the healing of periodontal tissue 19, muscle 20, and tendon injuries 21. Additionally, we compile clinical trials to provide a thorough understanding of the current potential of LIPUS in inflammation control and organ function restoration. It also concludes with a summary of its limitations and several prospects to promote further research in the field of LIPUS-mediated anti-inflammatory and reparative effects.

## 2. Therapeutic Ultrasound and LIPUS

Ultrasound is a form of mechanical characterized by sound pressure waves with frequencies beyond the limitation of human hearing (i.e. 20 to 20,000 Hz). It plays a vital role in modern medicine, serving not only as a diagnostic imaging modality but also as a therapeutic treatment 22. In therapeutic applications, ultrasound functions through both thermal and non-thermal mechanisms. The latter include ultrasonic cavitation, gas activation, mechanical stress, and other indeterminate non-thermal processes 23. Typically generated by piezoelectric sensors that convert electrical energy into mechanical force, LIPUS periodic waves propagate through the medium into target tissues. This causes vibration and impact, producing mechanical stimuli that promote biochemical events facilitating anti-inflammation and repair 10, 24, 25.

During this process, cavitation, acoustic streaming, and mechanical stimulation serve as the primary non-thermal effects of LIPUS, generating microbubbles and microjets to achieve therapeutic results 26. This non-thermal mechanism can also be explained by acoustic streaming effect during LIPUS exposure. Acoustic streaming may alter the local cellular microenvironment, influencing intracellular potassium and calcium content 27. These events impact the plasma membrane, focal adhesions, and cytoskeletal structures, thereby initiating intracellular signal transduction. Although the sensor molecules responsible for initially detecting these mechanical stimuli are not fully understood, we will discuss mechanosensitive structures that potentially play a role.

Generally, ultrasound is widely used in imaging medicine at intensities of 0.05-0.50W/cm^2^ 28. According to intensity, therapeutic ultrasound can be divided into two groups: low-intensity ultrasound (<3 W/cm^2^), and high-intensity ultrasound (≥3 W/cm^2^). Additionally, ultrasound is categorized into three ranges based on ultrasound frequency: high-frequency ultrasound (1-20 MHz) for medical diagnostic applications, medium-frequency ultrasound (0.7-3.0 MHz) for therapeutic medicine, and low-frequency (LF) ultrasound (20-200 kHz) for industrial and therapeutic applications 29. Typical pulse ratios of pulse duration to pulse rest time are 1:5 6.

The U.S. Food and Drug Administration (FDA) has approved therapeutic ultrasound for two main clinical areas: thermal applications (including physical therapy, thermotherapy, and high-intensity focused ultrasound for tissue ablation) and non-thermal applications (including extracorporeal shock wave lithotripsy, intracorporeal lithotripsy, and low-power kilohertz frequency devices) 22. LIPUS is a type of mid-frequency ultrasound (0.7-3 MHz) that is output in a pulsed-wave mode (100 and 1000 Hz) 30. Other parameters of LIPUS therapies include the ultrasound intensity ranging from 0.02-1 W/cm^2^ (spatial average temporal average, SATA) and a treatment duration of 5-20 minutes daily 31. Researchers typically use LIPUS at an intensity of 30 mW/cm² SATA, a frequency of 1.5 MHz, and a 1 kHz pulse repetition rate with a 20% duty cycle 30.

A growing number of clinical studies are currently investigating LIPUS therapy. For instance, in 2023, Li *et al.* 32 conducted a single-center, small-sample, exploratory clinical study on patients with COVID-19 pneumonia. Results showed improvements in C-reactive protein, interleukin-6, leukocytes, and fingertip arterial oxygen saturation (SaO2) after an average of 7.2 days of LIPUS treatment. This therapy reduced lung inflammation and serum inflammatory factor levels, attenuating pneumonia. Although LIPUS has demonstrated promising clinical results in anti-inflammation and repair, its specific mechanisms remain unclear. Recent studies have begun exploring these molecular mechanisms, providing a theoretical basis for broader clinical applications.

## 3. Biological and Molecular Mechanisms of LIPUS for anti-inflammation and repair

**Figure 1:**
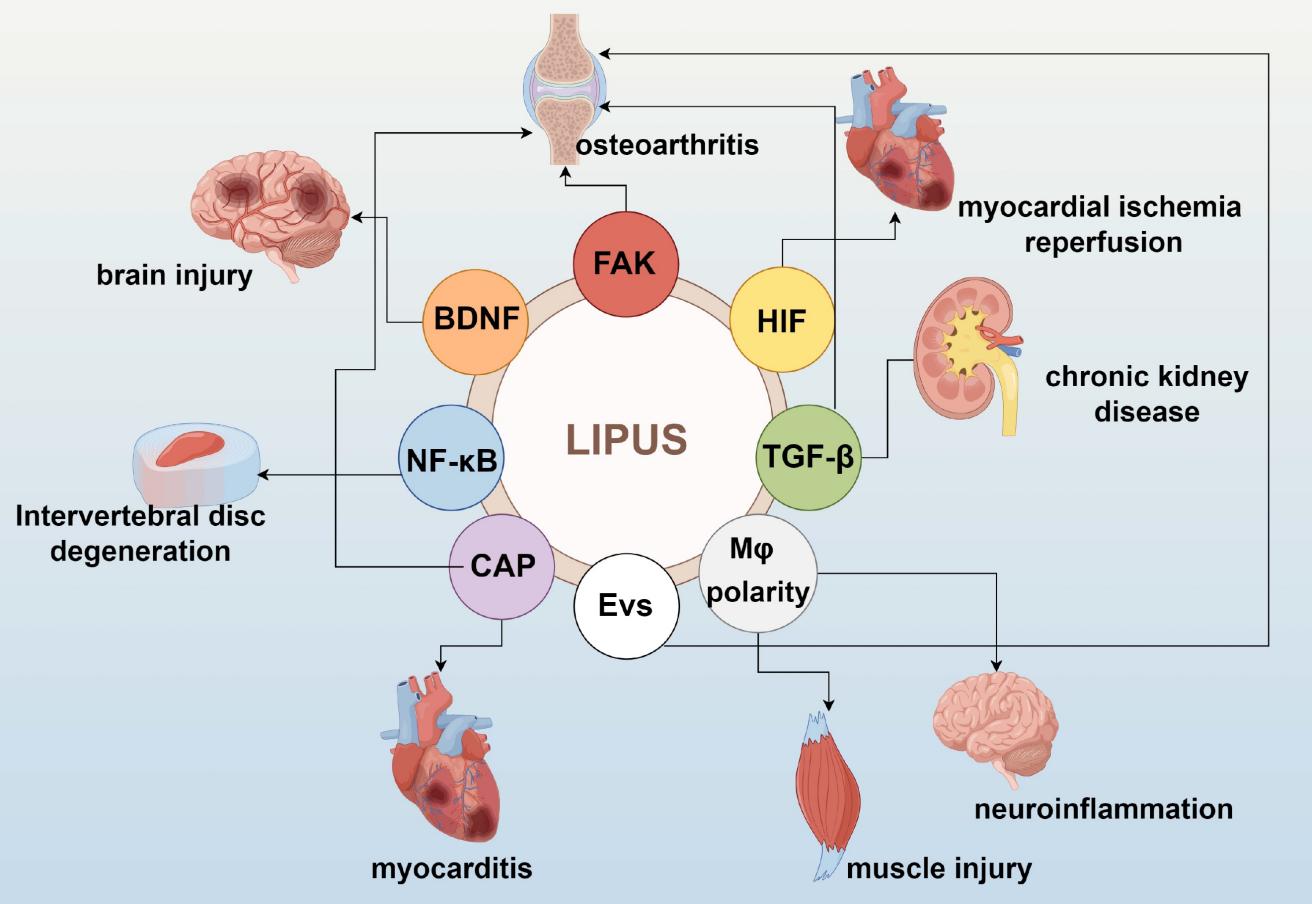
Molecular and cellular processes involved in the LIPUS bioeffects. By Figdraw. Abbreviations: BDNF: brain-derived neurotrophic factor; CAP: cholinergic anti-inflammatory pathway; EVs: extracellular vesicles; FAK: focal adhesion kinase; HIF: hypoxia-inducible factor; NF-κB: nuclear factor-κB.

**Figure 2:**
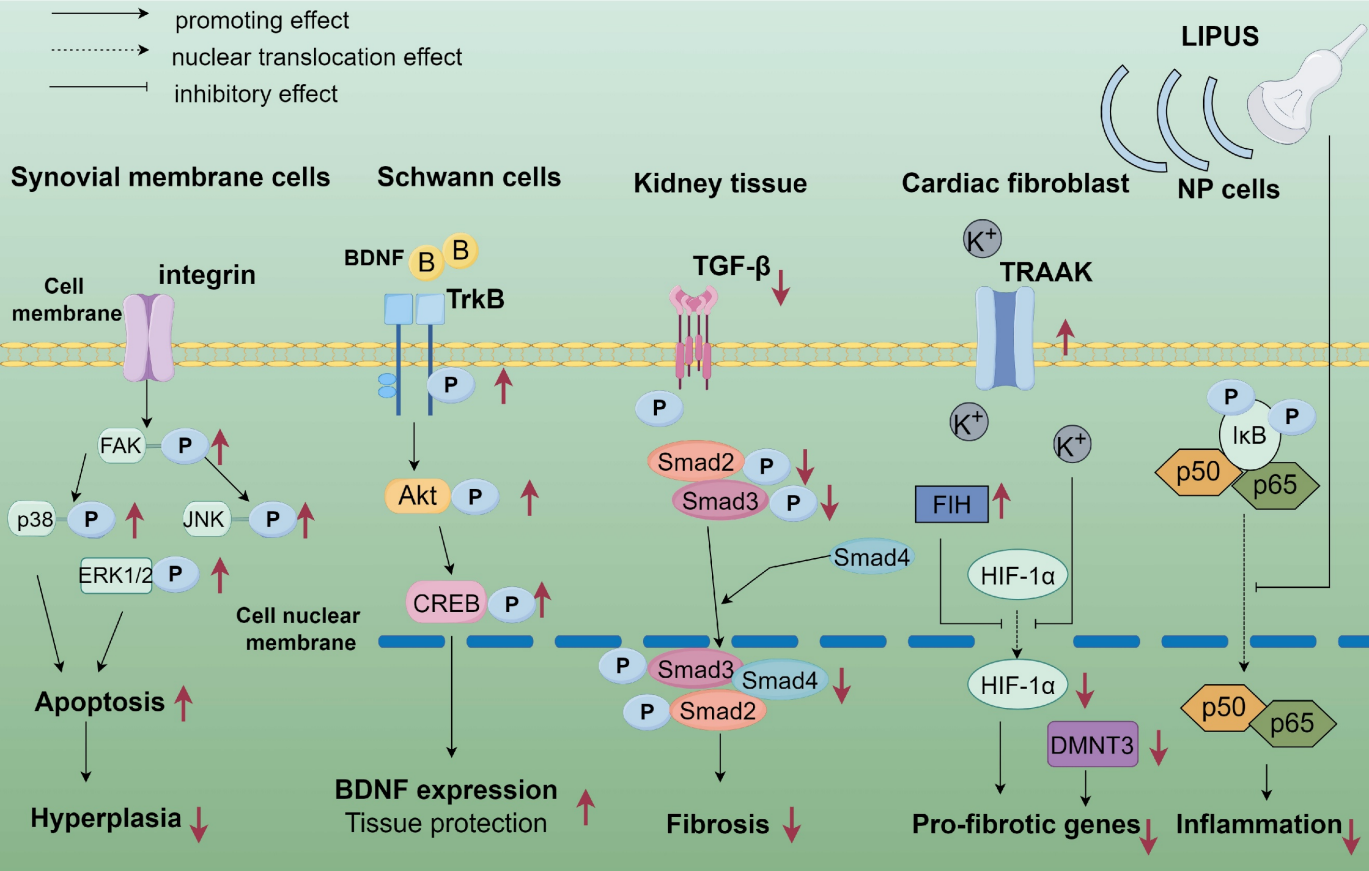
The application of LIPUS in anti-inflammation and repair at the biological and molecular level. LIPUS up-regulated phosphorylated FAK in synovial cells, leading to decreased ERK, c-JNK, and p38 phosphorylation. LIPUS modulated apoptosis and survival of osteoarticular synoviocytes through the integrin/FAK/MAPK signaling pathway. LIPUS activates SCs through the TrkB/Akt/CREB axis in peripheral nerves, enhancing SCs proliferation, migration, and nerve growth factor expression. In an adriamycin-induced chronic renal injury model, LIPUS treatment significantly ameliorated renal injury by blocking signaling between TGF-β1 and its downstream molecules, Smad2 and Smad3 to reduce fibrosis and inflammation. LIPUS partially mitigated hypoxia-induced cardiac fibroblast phenotypic changes through the TRAAK-mediated HIF-1α/DNMT3a signaling pathway. In LPS-induced NP cells, LIPUS inhibited the NF-κB signaling pathway by suppressing p-P65 protein expression and P65 translocation to the nucleus, i.e., inhibiting the NF-κB signaling pathway, inflammation, and catabolism in LPS-induced human degenerative medulla oblongata cells. By Figdraw. Abbreviation: CREB: cyclic-AMP response binding protein; DNMT3a: DNA methyltransferase3α; ERK: extracellular regulated protein kinases; HIF: hypoxia-inducible factor; JNK: c-Jun N-terminal kinase; LIPUS: low intensity pulse ultrasound; MAPK: mitogen-activated protein kinase; NF-κB: nuclear factor-κB; NP: nucleus pulposus; SCs: Schwann cells; TrkB: tyrosine Kinase receptor B; TGF-β: transforming growth factor-β; TRAAK: TWIK-related arachidonic acid-activated K^+^ channel.

**Figure 3:**
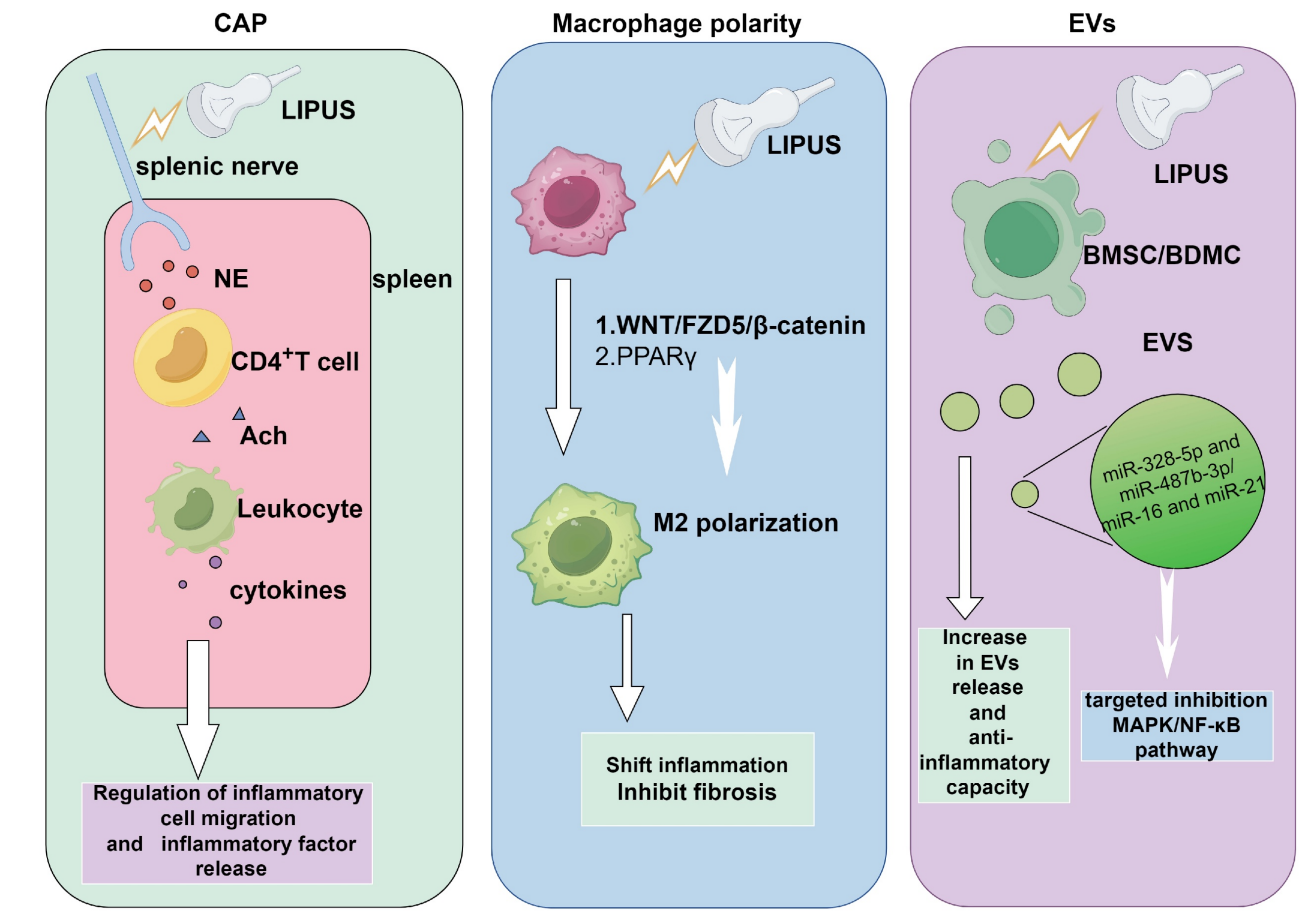
The capacity of LIPUS in anti-inflammation and repair via multiple signaling pathways. NE released from the splenic nerve treated by LIPUS stimulated CD4^+^ T cells to release ACh, making leukocytes releasing cytokines. This could modulate inflammatory cells migration and inflammatory factors release. EVs produced by LIPUS-treated BMSCs contained higher levels of miR-328-5p and miR-487b-3p, targeting genes in the MAPK signaling pathway. LIPUS treatment increased miR-16 and miR-21 levels in BMDC exosomes, targeting and inhibiting the NF-κB signaling pathway. The WNT/FZD5/β-catenin axis and PPARγ signaling pathway were encential for LIPUS to promote M2 polarization. By Figdraw. Abbreviation: Ach: acetylcholine; BMSCs, bone marrow-derived mesenchymal stem cells; BMDC: bone dendritic cell; CAP: cholinergic anti-inflammatory pathway; EVs: extracellular vesicles; LIPUS: low intensity pulse ultrasound; MAPK: mitogen-activated protein kinase; NF-κB: nuclear factor-κB; NE: Norepinephrine; PPARγ: peroxisome proliferator activated receptor γ.

LIPUS serves as a modulator of the tissue microenvironment and immune cell infiltration. Significant data from basic studies, both *in vivo* and *in vitro*, have accumulated regarding the role of LIPUS in anti-inflammation and repair. LIPUS exerts its anti-inflammatory and reparative effects by mechanically perturbing cells and tissues, triggering a cascade of biological responses. These include the activation of mechanosensitive pathways, regulation of inflammatory signaling, and modulation of immune cell phenotypes (Figure 1). In this section, we elucidate the biological and molecular applications of LIPUS in anti-inflammation and repair (Table 1) (Figure 2, 3).

The mechanical effects of LIPUS activate intracellular downstream pathways by affecting the structure of the cell membrane surface, the permeability of ion channels, and cell-cell and cell-matrix interactions 33. Recently, Piezo1 proteins have been identified as Ca^2+^ channels on various cell surfaces and play important roles as biomechanical transducers 34. In 2021, Zhang *et al.* 35 demonstrated that Piezo1 transduces LIPUS-associated mechanosignals into the cell via Ca^2+^ influx. This acts as a second messenger, activating extracellular signal-regulated kinase 1/2 (ERK1/2) phosphorylation and perinuclear F-actin polymerization to regulate the proliferation of mouse calvaria osteoblast-like cells. Furthermore, LIPUS stimulation increases cilia length in chondrocytes and affect the orientation of primary cilia in chondrocytes. This changes in cilia length and shape are reversible and may be associated with ERK1/2 activation 36. Direct cell-to-cell communication is also important for mechanotransduction 19. Another potential mechanism involves the initial activation of integrins in order to convert the mechanical energy of LIPUS 37.

LIPUS coordinates anti-inflammatory and repair responses through multiple molecular pathways. FAK, a non-receptor tyrosine kinase, plays a key role in integrin-mediated signaling, regulating cell adhesion migration, proliferation, and survival 38, 39. Sato *et al.* 19 reported that LIPUS transmits signals intracellularly via integrins acting as mechanoreceptors. LIPUS controls cell spreading on fibronectin through the FAK-Rab5-Rac1 pathway 40. Under LIPUS irradiation, FAK is phosphorylated via vinculin; subsequent FAK signaling leads to Rab5-dependent Rac1 activation, promoting actin cytoskeleton rearrangement and increased cell motility 40. Phosphorylation of FAK activated multiple signaling pathways by LIPUS exposure, such as integrin/FAK/mitogen-activated protein kinase (MAPK) 37, Src family kinases (SFKs) 41, FAK/phosphatidylinositol 3-kinase (PI3K)/Akt 42.

BDNF, synthesized in neurons and glial cells, is vital for neuronal differentiation, cell survival, and synaptic function in the nervous system 43. BDNF is sustainably released through a Ca^2+^ - dependent mechanism, binding strongly to tropomyosin receptor kinase B (TrkB) receptors to activate signaling pathways 44. LIPUS stimulation increased BDNF expression in astrocytes through TrkB/PI3K/Akt, TrkB/phospholipase C (PLC) - γ/Ca^2+^, and calcium/calmodulin stimulated protein kinase (CaMK) signaling pathways 45. BDNF levels can also be elevated through TrkB/Akt/CREB signaling pathway by LIPUS application 13.

Chronic inflammation, fibrosis, and cancer are common TGF-β-related diseases 46. LIPUS application generally attenuated fibrosis by inhibiting the TGF/Smad pathway 14, 24. Treatment with LIPUS blocks TGF-β1 and its downstream molecules, Smad2 and Smad3, as well as the nuclear factor erythroid 2-related factor 2/Kelch-like ECH-associated protein 1/heme oxygenase 1 (Nrf2/keap1/HO-1) pathway, thereby reducing fibrosis and inflammation 18.

Hypoxia-inducible factor (HIF) orchestrates cellular adaptation to low oxygen levels by controlling metabolic, angiogenic, and erythropoietic transcriptional programs 47. Given the heart's high oxygen demand for pumping blood, LIPUS holds promise for combating hypoxia-related inflammation 48. It reduces cardiac fibrosis by decreasing HIF-1α expression and suppressing ASK1 and JNK 10. Additionally, it protects against myocardial ischemia/reperfusion (MI/R) through the TWIK-related arachidonic acid-activated K^+^ channel (TRAAK)-mediated HIF-1α/DNA methyltransferase3α (DNMT3a) signaling pathway 17. By mitigating the hypoxic response, LIPUS reduces downstream harmful effects such as oxidative stress, inflammation, and fibrosis 17. This represents a direct molecular intervention at the level of hypoxia signaling, distinct from some therapies based on enhancing oxygen's physical diffusion 49.

The cholinergic anti-inflammatory pathway (CAP) is a neuro-immunomodulatory mechanism in the central nervous system that swiftly regulates systemic inflammatory responses. Norepinephrine (NE) released from the spleen stimulates CD4^+^ T cells to release acetylcholine (ACh), binding to the α7 nicotinic acetylcholine receptor (α7nAChR) on macrophages. The vagus nerve (VN) attenuates inflammatory responses in several systems by inhibiting splenic macrophages, including the lungs, digestive tract, myocardium, synovium, and kidneys 50. In colitis, Nunes *et al.* 51 reported that therapeutic ultrasound applied to the mouse abdomen activated the vagus nerve, splanchnic nerve, and CAP, inhibiting pro-inflammatory cytokine release. Furthermore, ACh released from enteric neurons to muscle macrophages in the intestine affected the colon and mesenteric lymph nodes (MLN), eliciting an anti-inflammatory response that alleviated dextran sodium sulfate (DSS)-induced colitis.

Regulating NF-κB transcriptional activity involves multiple mechanisms including ubiquitination, sumoylation, or phosphorylation of upstream and downstream mediators, IκB kinase (IKK) subunits, or NF-κB proteins themselves 52. Sato *et al.* 53 showed that LIPUS activates intracellular ubiquitin-editing protein A20 in response to inflammatory stimuli. This activates a negative feedback system in salivary gland acini and ductal cells to inhibit the NF-κB pathway and promote an anti-inflammatory response. Similarly, tumor necrosis factor (TNF) receptor associated factor 6 (TRAF6), an E3 ubiquitin ligase, activates the NF-κB pathway by linking to the K63-linked polyubiquitin chain, subsequently facilitating IKK complex activation 54. LIPUS induces the expression of F-box and leucine rich repeat protein 2 gene (FBXL2), mediates TRAF6 ubiquitination, reduces TRAF6 and NF-κB levels and inhibits the downstream inflammatory signaling pathway 55. Additionally, LIPUS suppresses p-P65 protein expression and P65 translocation to the nucleus, inhibiting the NF-κB pathway 56.

LIPUS directly modulates immune cell behavior and optimizes the microenvironment. Activated macrophages are typically classified into M1 and M2 types, influenced by factors such as microbes, the tissue environment, and cytokine signals. Excessive M1 activity drive chronic inflammation and inflammatory diseases. M2 macrophages are key to tissue repair and immune tolerance 57. Rational spatiotemporal modulation of macrophage polarization can be achieved through LIPUS, reducing inflammation and inhibiting fibrosis 58. Moreover, continuous LIPUS mechanical force suppressed toll-like receptor 4 (TLR4) mRNA levels in LPS-induced macrophages and inhibited degradation and phosphorylation of IκBα and p65 nuclear translocation 59. Notably, TLR4 protein expression showed no significant difference with or without LIPUS, suggesting a possible impact on TLR4 gene transcription rather than direct down-regulation of the existing receptor. Zhao *et al.* 55 found that LIPUS induces the expression of F-box/FBXL2, reduces TRAF6/NF-κB levels and inhibites the downstream inflammatory signaling pathway in macrophage.

Extracellular vesicles (EVs) are membrane-encapsulated particles produced by nearly all cell types that serve as a pathway for intercellular communication 60. The increased production of LIPUS-mediated EVs, along with the greatly enhanced anti-inflammatory and repair capabilities, holds great promise for the engineered production and application of EVs. Li *et al.* 61 demonstrated that LIPUS stimulation increased EVs release from BMSCs by 3.66-fold. It promoted proliferation, EVs secretion, and anti-inflammatory functions of BMSCs without affecting cell morphology and apoptosis. EVs produced by LIPUS-treated BMSCs (LIPUS-EVs) contained higher levels of miR-328-5p and miR-487b-3p compared to those from non-LIPUS-treated BMSCs and exerted anti-inflammatory functions by targeting genes in the MAPK signaling pathway. When cultured with RAW264.7 cells, LIPUS-EVs induced higher anti-inflammatory factor (IL-10) expression and lower NF-κB signaling activity in response to LPS. *In vivo*, mice injected with LIPUS-EVs exhibited higher allogeneic skin IL-10 expression, lower pro-inflammatory factor (IL-6) expression, and reduced immune cell infiltration. Thus, both *in vitro* and *in vivo* experiments demonstrate that LIPUS enhances the anti-inflammatory effects of BMSC-derived EVs. Similarly, Li *et al.* 62 found that LIPUS treatment increased miR-16 and miR-21 levels in barrow bone dendritic cell (BMDC) exosomes, targeting and inhibiting the NF-κB signaling pathway.

## 4. Application of LIPUS for anti-inflammation and repair

**Figure 4:**
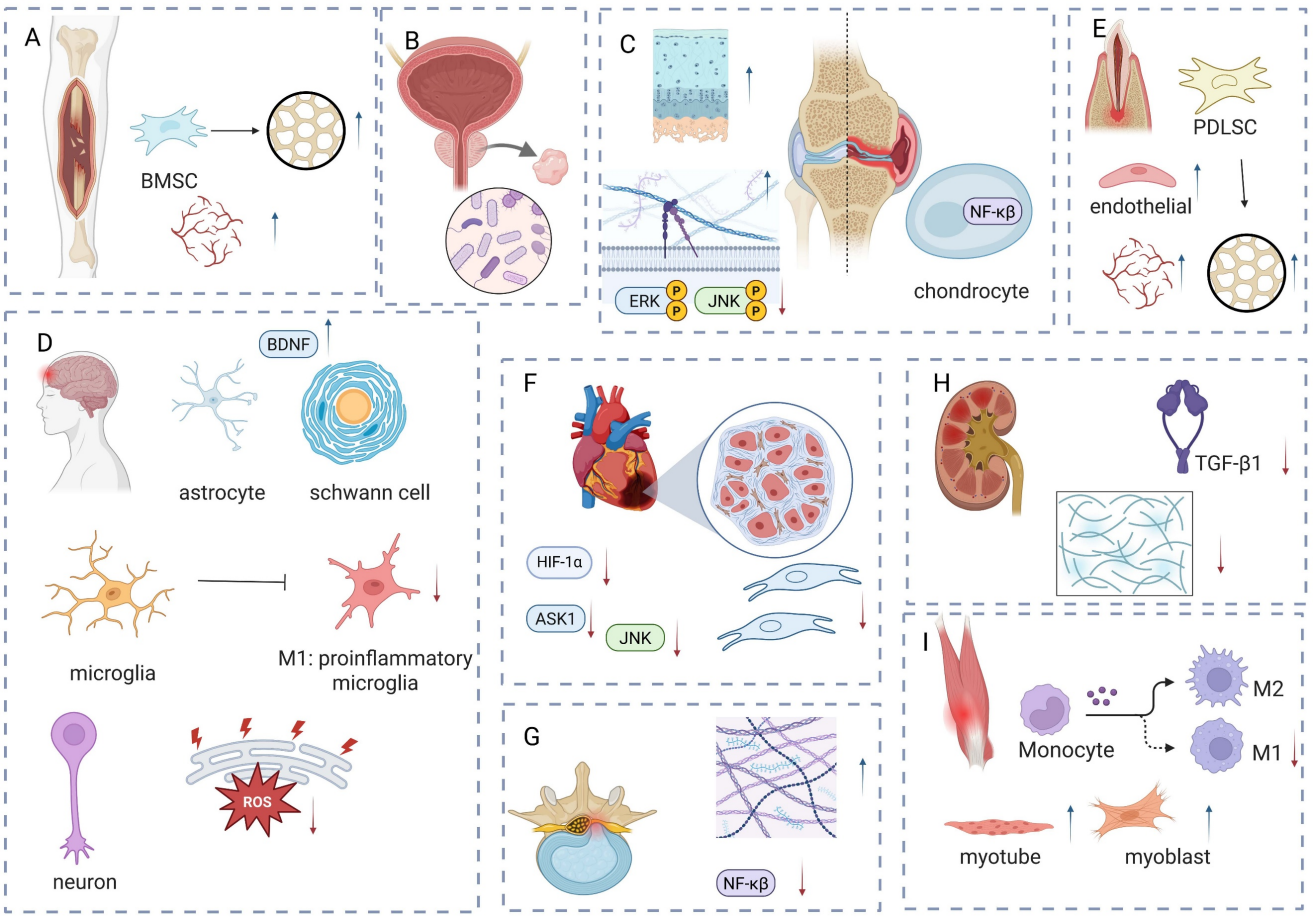
The main mechanism of LIPUS in tissue injury: anti-inflammation and repair. LIPUS promotes the osteogenic differentiation of BMSCs and enhances angiogenesis to facilitate fracture healing. (B) LIPUS improves chronic prostatitis by regulating the balance of microbiota. (C) LIPUS reduces inflammation in chondrocytes and synovial cells, while promoting the formation of the extracellular matrix. (D) LIPUS promotes the increase of BDNF to protect the brain or peripheral nerve injury and reduce the infiltration of M1-like inflammatory cells; it alleviates ER stress to protect neurons. (E) LIPUS promotes the increase of BDNF to protect the brain or peripheral nerve injury and reduce the infiltration of M1-like inflammatory cells; it alleviates ER stress to protect neurons. (F) LIPUS reduces the production of inflammatory factors and fibroblasts to alleviate inflammation and fibrosis. (G) LIPUS alleviates degenerative human nucleus pulposus by promoting extracellular matrix formation and reducing inflammatory factors. (H) LIPUS alleviates renal injury-induced fibrosis by reducing TGF. (I) LIPUS promotes muscle injury repair by reducing M1 cells and increasing myotubes and myoblasts. Figure was drawn by BioRender.com. Abbreviation: BDNF: brain-derived neurotrophic factor; BMSC: bone marrow-derived mesenchymal stem cell; HIF: hypoxia-inducible factor; HPDLC: human periodontal ligament cell; JNK: c-Jun N-terminal kinase; LIPUS: low intensity pulse ultrasound; NF-κB: nuclear factor-κB; TGF-β: transforming growth factor-β.

LIPUS has been used clinically in various contexts, with an increasing number of clinical trials and animal experiments revealing its potential for use in anti-inflammation and repair (Table 2, Figure 4). In inflammation-related diseases such as chronic prostatitis, osteoarthritis, cardiovascular diseases, and renal injuries, LIPUS can alter microbial communities, regulate cell metabolism and signaling pathways, and inhibit inflammation and fibrosis to exert therapeutic effects. In neuro-related disorders such as brain dysfunction and peripheral nerve injuries, LIPUS promotes neurogenesis, regulates cell activity, and gene expression to support nerve repair. It also aids in repair periodontal tissue damage, muscle, and tendon injuries by regulating cell differentiation and intercellular communication.

### 4.1 Bone fracture

In 1994, the FDA approved LIPUS for clinical use in the repair of fresh fractures 63. In 2017, Leighton R *et al.* reported a healing rate of over 80% for LIPUS treatment of 1441 cases of non-union fractures (at least 3 months old) at various anatomical sites 64. Padilla *et al.* and McCarthy *et al.* reviewed the application of ultrasound in bone repair, focusing on LIPUS. They discussed dosage standardization and the potential thermal and non-thermal biological effects on bone healing, including effects on gene expression, signaling molecules, mechanotransduction pathways, and the extracellular environment 65, 66.

Yao *et al.* 67 prepared nanomechanical force generators and explored their combined effects with LIPUS on osteogenesis in BMSCs. LIPUS was applied at 3 MHz frequency, 50% duty cycle, and appropriate intensity of 100mW/cm^2^. The combination of LIPUS and nanomechanical force generators promotes osteogenesis and bone formation in BMSCs by modulating transient receptor potential melastatin 7 (TRPM7), the actin cytoskeleton, and intracellular calcium oscillations. The specific mechanism involves nanomechanical force generators binding to integrin receptors on the BMSC membrane, transmitting LIPUS mechanical effects, and inducing changes in TRPM7 activity to enhance extracellular calcium influx.

A study on 40 patients with anterior mandibular fractures assessed the effect of LIPUS on healing. Patients were randomly assigned to a control group (20 cases) or a treatment group (20 cases), both receiving standard surgery. The treatment group received LIPUS (1.5 MHz, 30 mW/cm², 20 minutes daily) on days 4, 8, 14, and 20 post-surgery. Results showed significantly better pain relief, wound healing, and fracture healing scores in the treatment group. At 12 weeks, 60% of the treatment group had complete healing, compared to 15% in the control group 68. In another study on delayed fibular fracture healing, 13 patients (9 women, 4 men) were divided into LIPUS treatment (7 cases) and control groups (6 cases). LIPUS was applied daily for 20 minutes with parameters of 1.5 MHz frequency, 1 kHz modulation, and 30 mW/cm^2^ peak pressure for 5 months. Histological and morphometric analysis showed that LIPUS increased small blood vessels in the newly formed bone, with a 70% increase in vessel size and trends of increased vascular density 69.

### 4.2 Chronic prostatitis

LIPUS has also shown promise in treating of chronic inflammation, such as chronic prostatitis/chronic pelvic pain syndrome (CP/CPPS) 70. In 2013, Li *et al.* evaluated the clinical efficacy and safety of transperineal ultrasound therapy for CP treatment. Analyzing the National Institutes of Health Chronic Prostatitis Symptom Index (NIH-CPSI) scores, routine prostate exams and prostate fluid expression in a randomized, double-blind, multicenter trial of 96 patients, results showed that the overall efficacy of 70.83% in the LIPUS group 71.

LIPUS is gaining attention as a novel physical therapy for refractory type IIIB prostatitis. It may exert therapeutic effects by altering the microbial community structure in prostatic fluid. In a study of 25 patients with type IIIB prostatitis, LIPUS treatment (10 minutes per session, every other day, 5 sessions per cycle, two cycles) reduced NIH-CPSI scores by 4 points or more. Song *et al.* found that LIPUS significantly affected the bacterial composition in prostatic fluid, reducing pathogens such as *Veillonella*, *Methyloversatilis*, and *Ureaplasma*. The proposed mechanism suggests the ultrasound energy disrupts bacterial cell structures, reducing their numbers and alleviating prostatitis symptoms. Additionally, changes in microbial diversity suggest that LIPUS may improve the inflammatory environment by restoring microbial balance 15.

### 4.3 Osteoarthritis

Zhang *et al.* 41 demonstrated that LIPUS enhances cartilage healing through the phosphorylation of FAK and Src family kinases (SFKs). The complex formation induces chondrogenic progenitor cells (CPCs) to migrate to the cartilage injury site, delaying or preventing post-traumatic osteoarthritis (PTOA) onset. Furthermore, LIPUS accelerated cartilage matrix synthesis to promote cartilage regeneration. Peng *et al.* 37 reported that LIPUS regulates extracellular matrix expression in early and late osteoarthritic cartilage through the integrin/FAK/MAPK pathway. While LIPUS significantly increased type II collagen expression in early osteoarthritic cartilage, it decreased matrix metalloproteinase-13 (MMP-13) levels. LIPUS regulates chondrocyte metabolism by stimulating the primary cilia, which activate TRPV4 channels and modulate intracellular Ca^2+^ levels. This suppresses endoplasmic reticulum stress, reduces chondrocyte apoptosis, and increases the expression of cartilage matrix components. Additionally, silencing IFT88, which affects primary cilia expression, disrupts LIPUS's protective effect on cartilage 72.

Furthermore, impaired autophagy leads to yes associated protein (YAP) accumulation, which binds with receptor-interacting protein kinase 1 (RIPK1), to activate the NF-κB pathway, exacerbating chondrocyte inflammation and extracellular matrix degradation. LIPUS inhibits this progression by restoring autophagy and reducing YAP-RIPK1 interaction. Optimal effects were achieved at 30 mW/cm² for 20 minutes, significantly suppressing inflammation and promoting matrix homeostasis 16. Liao *et al.* 73 injected exosomes derived from bone marrow mesenchymal stem cell (BMSC) treated by LIPUS into the joint cavity. This treatment significantly attenuated cartilage destruction and promoted cartilage regeneration in OA. Furthermore, LIPUS irradiation of BMSC notably increased exosome production, enhancing their capacities.

LIPUS reduces infiltration and inflammation in the synovium. Osteoarticular synoviocytes may undergo modulation of apoptosis and survival through the integrin/FAK/mitogen-activated protein kinase (MAPK) pathway. Sato *et al.* 74 found that LIPUS upregulates phosphorylated FAK in synovial cells, leading to decreased extracellular regulated protein kinases (ERK), c-Jun N-terminal kinase (JNK), and p38 phosphorylation. Zachs *et al.* 75 investigated upregulated genes in T and B cells of arthritic mice treated with LIPUS-stimulated spleen. Changes in gene expression affecting cytoskeletal regulation influenced lymphocyte polarization and migration, reducing synovial infiltration. In addition, increased vagal signaling led to the aggregation of B cells in the splenic marginal zone. Genes encoding transcriptional regulators such as c-Jun and JunB were upregulated in T and B cells, potentially influencing inflammatory pathways. To address inflammation-induced fibrosis, Zhou *et al.* 24 observed that LIPUS alleviated immobilization-induced knee joint capsule fibrosis by inhibiting reactive oxygen species (ROS) production and activation of the TGF-β1/Smad signaling pathway.

LIPUS stimulation of rat mandibular chondrocytes in hypoxic conditions increased HIF-1α expression while decreasing HIF-2α 48, alleviating hypoxia-induced chondrocyte damage. Nagao *et al.* 76 demonstrated that LIPUS mechanical stress on angiotensin II receptor type 1 (AT1) activates phospholipase C-β (PLCβ). This mediates anti-inflammatory effects in LPS-induced osteoblasts by inhibiting NF-κB activation and decreasing IL-1α production. Moreover, the combination of LIPUS (1.5 MHz, 80 mW/cm^2^) and Fe^3+^, significantly accelerates the proliferation and differentiation of osteoblasts compared to single-factor treatments 72. In osteoarthritis repair, LIPUS improves the trabecular microstructure and histological features of subchondral bone by inhibiting osteoclast activity and the TGF-β1/Smad3 signaling pathway in a temporomandibular joint osteoarthritis (TMJOA) rabbit model 14.

### 4.4 Brain damage and dysfunction

LIPUS exposure increased BDNF expression in astrocytes in both *in vitro* and *in vivo* rat models, enhancing BDNF production through TrkB/PI3K/Akt, TrkB/phospholipase C(PLC)-γ/Ca^2+^, and calcium/calmodulin stimulated protein kinase (CaMK) signaling pathways 45. BDNF/TrkB signaling can further boost BDNF induction via cAMP response element-binding protein (CREB) through PI3K/Akt or ERK signaling 77. Su *et al.* 78 reported that LIPUS therapy after traumatic brain injury (TBI) promoted BDNF production through the TrkB/Akt/CREB signaling pathway. This increased vascular endothelial growth factor (VEGF) and reduced cleaved caspase-3 levels to prevent apoptosis. Wang *et al.* 79 applied LIPUS on TBI (800 kHz, 3 W/cm², 10 minutes daily), finding it promotes hippocampal neurogenesis, increases newborn neuron counts, and enhances phosphatidylcholine levels, thereby improving neural activity and cognitive function. Mechanisms may involve metabolic regulation and gene expression related to neurogenesis.

LIPUS inhibited the inflammatory response in activated microglia by modulating the signal transducer and activa tor of transcription 1 (STAT1)/STAT6 (attenuating STAT1 phosphorylation and promoting STAT6 phosphorylation) and peroxisome proliferator activated receptor γ (PPARγ) signaling pathways 80. This reduces M1 markers (cytotoxic nitric oxide) and promotes M2 markers (L-arginine), shifting the phenotype to favor reduced cytotoxicity. LIPUS boosted M2a and M2c markers (CD206 and TLR8) but not the M2b marker CCL1. Unlike many drugs, LIPUS stands out due to its ability to penetrate the blood-brain barrier 80. Li *et al.* 81 focused on LIPUS effects on cortical microelectrode implantation (1.13 MHz, 300 mW/cm²). LIPUS significantly accelerates the migration of microglia in the early post-implantation, promoting a branched morphology, and enhances monitoring activity. In later stages, it reduces microglial coverage around the electrode and astrocytic scar formation, while improving neuronal recording performance. Potential mechanisms involve ATP release, metabolic regulation. In heart failure rats, head stimulation with LIPUS alleviated neuroinflammation and sympathetic overactivation by inhibiting the P2X7/NLRP3 pathway in microglia, preventing ventricular arrhythmias 82. LIPUS stimulation (100 mW/cm^2^) increases hippocampal neuronal cytosolic Ca^2+^ concentration within 5 minutes by enhancing Ca^2+^ influx through L-type calcium channels (LTCCs), without effecting stored Ca^2+^. It also promotes CREB phosphorylation and nuclear translocation, dependent on LTCC-induced Ca^2+^ influx 83. Truong *et al.* 84 showed that LIPUS reduces endoplasmic reticulum (ER) stress in motor neuron cells by lowering toxic protein levels. It protects cells from oxidative stress and mitochondrial dysfunction by reducing ROS and increasing mitochondrial membrane potential. LIPUS activates calcium signaling, AKT, and calcium-dependent transcription factors, enhancing cell survival (1.15 MHz, 356.78 mW/cm², 8 minutes). A protocol was established to investigate wearable LIPUS for Parkinson's Disease (PD) motor symptoms 85. This was a single-center, prospective, double-blind, randomized controlled trial, aiming to recruit 48 PD patients. The LIPUS group receives treatment (600 kHz, 1.0 W/cm², 50% duty cycle) for 30 minutes daily over 4 months, compared to a sham group.

### 4.5 Peripheral nerves injury

Xia* et al.* 19 reported that LIPUS significantly upregulated phosphorylated FAK and activated ERK1/2 to promote proliferation and neural differentiation of iPSCs-NCSCs, aiding peripheral nerves regeneration. Furthermore, LIPUS activated Schwann cells (SCs) through the TrkB/Akt/CREB axis in peripheral nerves. This treatment enhanced SCs proliferation, migration, and nerve growth factor expression, effectively treating erectile dysfunction (ED) resulting from bilateral cavernous nerve injury 13.

Exosomes from LIPUS-stimulated SCs (LIPUS-SCs-Exo) showed the surprising capacity to enhance axonal growth of pelvic ganglion (MPG) neurons/cavernous nerves and MPG neurons. In rats with bilateral cavernous nerve crush injury (BCNI) induced ED, LIPUS-SCs-Exo promoted injured CN regeneration and SCs proliferation. High-throughput sequencing showed that LIPUS stimulation regulates the genes of MPG neurons by altering SCs-Exo derived miRNAs, thereby activating the PI3K/Akt/Forkhead Box O (FoxO) signaling pathway, promoting nerve regeneration and restoring ED 86.

### 4.6 Periodontal tissue damage

During orthodontic treatment, mechanical forces trigger alveolar bone changes. LIPUS plays a crucial regulatory role in this process. LIPUS plays a crucial regulatory role in this process. Hu *et al.* 25 found that LIPUS promoted endothelial differentiation and angiogenesis of periodontal ligament stem cells (PDLSCs) through Piezo1 activation, promoting tissue regeneration. LIPUS exposure might induce osteogenic differentiation of PDLSCs through the BMP-Smad signaling pathway 87. This irradiation significantly increased Smad1/5/8 phosphorylation, leading to its subsequent translocation from the cytoplasm to the nucleus. Zhong *et al.* 88 demonstrated that LIPUS mitigated alveolar bone resorption under pressure and enhanced osteogenic capacity by downregulating Piezo1 expression.

LIPUS regulated osteoblast-osteoclast crosstalk for orthodontic alveolar bone remodeling. In 2023, Zhou *et al.* 33 reported that in a BMSC and bone marrow mononuclear cell (BMM) co-culture, the EphrinB2/EphB4 dimer exerted mechanotransduction functions in response to LIPUS. It regulated YAP nuclear translocation through F-actin rearrangement, facilitating osteogenesis and alveolar bone remodeling.

### 4.7 Cardiovascular disease

Cao *et al.* 10 revealed that hypoxia during MI/R triggered nuclear HIF-1α binding, upregulates NADPH oxidase 4 (Nox4), and increased ROS, leading to inflammation. LIPUS (0.5 W/cm²) attenuated this response by decreasing HIF-1α expression and suppressing ASK1 and JNK pathways. Sun *et al.* 89 used LIPUS (1 MHz, 0.25 W/cm2 and 20% duty ratio) to treat the MI/R mouse model. The LIPUS group received treatment for 20 min three times a day on days 0-7, and twice a week during weeks 2-4, and a duty cycle of 20%. LIPUS activates the RhoA/Myosin II pathway to promote cytoskeletal rearrangement and migrasome formation. It also activates YAP nuclear translocation, which in turn transcriptionally activates KIF5B and Drp1 to facilitate mitocytosis.

Liu *et al.* 90 observed that LIPUS significantly increased Treg cell infiltration in myocarditis hearts and decreased the migration of pro-inflammatory CCR2^+^ macrophages. LIPUS modulated inflammatory cytokines associated with Th17 cells, Treg cells, and macrophages. Besides alleviating inflammation, LIPUS can mitigate hypoxia-induced cardiac fibrosis. Zhao *et al.* 17 demonstrated that LIPUS partially mitigated hypoxia-induced cardiac fibroblast phenotypic changes through the TWIK-related arachidonic acid-activated K^+^ channel (TRAAK)-mediated HIF-1α/DNA methyltransferase3α (DNMT3α) pathway. It reduced fibrotic markers like α-SMA, TGF-β, and collagen I. However, intensity matters: while 0.5 W/cm² provided protection, 2.5 W/cm² worsened cardiomyocyte injury 10.

LIPUS can transmit signals integrins 19. Bernal *et al.* 9 found that in cardiac mesoangioblasts (CMA), LIPUS triggers a conformational change in β1-integrin to its active form, promoting BMP receptor activation. Shindo *et al.* 91 evaluated the efficacy and safety of LIPUS in 64 patients with refractory angina. During LIPUS treatment, patients were positioned in the left lateral decubitus, with the probe placed on the anterior chest wall. Treatment was applied at a frequency of 1.875 MHz, intensity of 0.25 MPa, and 32 cycles. Each section was irradiated for 20 minutes, with a 5-minute interval between sections. Treatments were administered every other day, for a total of 3 days. While both LIPUS and placebo groups showed reduced nitroglycerin usage, there was no significant difference between them. However, the all-cause mortality rate was lower in the LIPUS group (3.1%) vs. placebo (13.8%), though not statistically significant.

In diabetic wound healing, LIPUS enhances the therapeutic effects of adipose-derived stem cell exosomes (ADSC-Exos). *In vivo*, LIPUS increased exosome uptake, prolonged their retention at the wound site, and promoted angiogenesis and wound healing. *In vitro,* LIPUS enhanced exosome uptake by 10.93 times and significantly promoted human umbilical vein endothelial cells (HUVECs) proliferation, migration, and tube formation 92. A study on 25 patients with Buerger's disease showed that LIPUS (2.0 MHz, 30 mW/cm², 20 minutes daily, 20% duty cycle) significantly reduced resting pain and improved skin perfusion pressure over 24 weeks 93.

### 4.8 Degenerative human nucleus pulposus

Zhang *et al.* 42 discovered that LIPUS promotes extracellular matrix (ECM) synthesis in degenerative human nucleus pulposus (NP) cells using the FAK/phosphatidylinositol 3-kinase (PI3K)/Akt pathway. Yi *et al.* 56 reported that in LPS-induced NP cells, LIPUS inhibited the NF-κB pathway by suppressing p-P65 protein expression and P65 nuclear translocation, blocking inflammation and catabolism.

### 4.9 Kidney injury

Inoue *et al.* demonstrated that ultrasound therapy before ischemia-reperfusion injury preserved kidney structure and function through CAP activation 94. Similarly, in an adriamycin-induced chronic renal injury model, LIPUS ameliorated injury by blocking TGF-β1/Smad signaling and the Nrf2/keap1/HO-1 pathway to reduce fibrosis and inflammation 18.

### 4.10 Muscle and tendon injury

Evaldo *et al.* 20 used LIPUS to treat cryoinjury in rat tibialis anterior muscle. LIPUS decreased neutrophils and inflammatory macrophages (M1) in the first 24 hours. By day two, reparative macrophages (M2) increased, but by day three, M2 levels were lower than in untreated muscles. This suggests LIPUS promotes the early transition of inflammatory macrophages and regulates reparative macrophages to attenuate fibrosis. Qin *et al.* 58 demonstrated that the WNT/FZD5/β-catenin axis was key for LIPUS-promoted M2 polarization. Macrophage depletion studies confirmed their integral role in LIPUS-mediated effects 95.

Duan *et al.* 96 highlighted the beneficial effects of LIPUS treatment on skeletal muscle regeneration, primarily through the modulation of satellite cell/myoblast mitochondrial metabolism. LIPUS (1 MHz, 250 mW/cm², 20% duty cycle) accelerated recovery, increased satellite cell counts, and reduced fibrosis via the AMP-activated protein kinase pathway. In rotator cuff repair, Wu *et al.* 21 investigated exosomes from BMSCs pretreated with LIPUS. These exosomes promoted fibrous cartilage regeneration at the bone-tendon interface, reduced fat infiltration, and regulated specific miRNAs (e.g., miR-140).

## 5. Discussion and conclusion

LIPUS has emerged as a highly promising non-invasive physical therapy with the potential to address a diverse range of inflammatory diseases and tissue repair needs. It has demonstrated significant capabilities in anti-inflammation and promoting tissue repair across multiple conditions, including bone fractures 69, chronic prostatitis 26, 70, osteoarthritis 48, 75, brain damage 78, peripheral nerve injuries 13, 19, periodontal tissue damage 87, cardiovascular diseases 9, 10, kidney injuries, muscle and tendon injuries 96, and degenerative human nucleus pulposus 56.

In the realm of basic research, numerous *in vivo* and *in vitro* studies have elucidated several key molecular mechanisms underlying LIPUS's effects. These involve the modulation of signaling pathways such as FAK, BDNF, TGF-β, HIF, CAP, NF-κB, and the regulation of macrophage polarization and extracellular vesicles. These pathways are not isolated; rather, they are interconnected, forming a complex bioregulatory network. For instance, LIPUS can stimulate BDNF expression in astrocytes directly 78 or via NF-κB activation 45. The NF-κB signaling pathway can be targeted and inhibited by LIPUS-EVs 61 or directly irradiation 56. Compared to targeted drugs, such as those that specifically inhibit TLR8 or target the MAPK/NF-κB pathway, LIPUS, as a physical therapy, offers a safe, multi-functional technology particularly suited for localized inflammation and tissue repair. Molecularly targeted drugs, on the other hand, provide powerful precision capabilities, making them ideal for systemic or intractable diseases 97, 98.

As we also mentioned, LIPUS regulates macrophage migration activation, the TGF-β/SMAD signaling pathway and HIF-related molecules 17, 24, 90. Recent findings suggest that Ly6C^hi^ macrophages migrate to the hypoxic zone and secrete oncostatin-M, in a HIF-1α-dependent manner, inhibiting TGF-β/SMAD signaling 99. This indicates a potential regulatory network for LIPUS in preventing cardiac fibrosis.

Different combinations of intracellular signaling pathways may produce varying effects depending on cell state, LIPUS intensity, and duration. For example, LIPUS effectiveness in osteoarthritis varies; while it improves cartilage function in mild cases, its effect may be limited in severe cases 37. While efficacy is well-demonstrated in preclinical studies, clinical data remain limited. A systematic review of bone traction osteogenesis found that LIPUS did not significantly shorten treatment duration or affect complications 100. Therefore, high-quality clinical trials are needed to validate translational potential. Standardized parameters for LIPUS use must be established, and adverse reactions monitored.

Although numerous signaling pathways (e.g., FAK, BDNF, TGF-β, NF-κB) have been identified, the current understanding of LIPUS efficacy should not be viewed as a collection of isolated events. Instead, LIPUS likely functions by orchestrating a “unified biological picture” through the spatiotemporal coupling of mechanotransduction and inflammatory signaling. The mechanical signals initiated by LIPUS are not merely physical perturbations; they act as upstream switches that modulate downstream chemical signaling networks. For instance, a critical crosstalk likely exists between mechanosensitive ion channels (e.g., Piezo1) and classical inflammatory pathways (e.g., NF-κB). Mechanical deformation of the cell membrane by LIPUS opens calcium channels, and the resulting Ca^2+^ influx may act as a temporal regulator-brief, controlled influxes can activate reparative pathways like ERK1/2 and BDNF, while potentially inhibiting the sustained inflammatory activation of NF-κB. This suggests that LIPUS acts as an “intelligent” modulator, shifting the cellular state from a pro-inflammatory phenotype to a reparative one by integrating physical cues with biochemical responses. Future research must decipher this spatiotemporal coupling: how the immediate physical effect on the membrane translates into delayed, long-term gene expression changes that resolve inflammation.

A critical analysis of the dose-effect relationship remains a significant gap in current literature (Table 3). The biological outcome of LIPUS is strictly parameter-dependent, where “more” is not necessarily “better”. Variations in frequency, intensity, and duty cycle can lead to divergent, sometimes opposite, biological results. Tissue type and parameters influence context-dependent responses. For example, in bone repair, LIPUS can promote osteogenesis through pathways related to integrins and calcium oscillations 67. In osteoarthritis, it can regulate chondrocyte metabolism and extracellular matrix synthesis 16, 37, and in brain disorders, it can enhance neurogenesis and modulate microglia activation 80. While lower intensities (e.g., 30-100 mW/cm²) typically promote anti-inflammatory M2 macrophage polarization and tissue regeneration 30, 58, higher intensities (e.g., >2.5 W/cm²) can induce thermal damage, exacerbate oxidative stress, or trigger cardiomyocyte injury 10. Frequency also dictates the depth of penetration and the scale of interaction; lower frequencies (kHz range) may generate different acoustic streaming patterns compared to MHz frequencies, affecting how mechanoreceptors are triggered. Consequently, there is no “one-size-fits-all” protocol. We propose that future optimization should move towards “disease-specific parameter windows”. For superficial, acute inflammation (e.g., wound healing), lower intensities with higher frequencies might suffice to stimulate angiogenesis without aggravating inflammation. Conversely, deep-tissue chronic fibrosis might require distinct modulation patterns to penetrate effectively while inhibiting TGF-β signaling. Establishing a standardized matrix of parameters correlated with specific biological endpoints (e.g., ROS levels, cytokine profiles) is essential for clinical reproducibility.

## 6. Perspective

Future research on LIPUS should focus on several key areas. Studies must transcend tissue-level observations by leveraging single-cell multi-omics, spatial transcriptomics, and epigenomics. This will enable the precise identification of cell subpopulations regulated by LIPUS, revealing their roles in reprogramming cell fate and intercellular communication. Constructing pseudo-temporal trajectories will help map the complete pathway of LIPUS-promoted anti-inflammation and repair. First, it is crucial to investigate the intricate relationships and synergistic effects among signaling pathways. Understanding these interactions could provide deeper insights into the mechanism of action. Second, clinical translation can be advanced by integrating LIPUS with advanced biomaterials. Developing LIPUS-responsive smart drug delivery systems enables the synergistic release of physical stimulation and chemical drugs. Combining LIPUS with tissue engineering scaffolds can construct biomimetic microenvironments to guide cellular behavior. Efforts must be made to establish standardized parameters (intensity, frequency, duration) for specific diseases. Standardization will enhance reproducibility and facilitate bench-to-bedside translation. Finally, comprehensive clinical trials with larger sample sizes and long-term follow-up are essential to accurately assess safety and efficacy. Comparative studies will determine the most effective treatment strategies.

## Figures and Tables

**Table 1:** The application of LIPUS in anti-inflammation and repair *in vivo* and *in vitro* studies

Mechanism	Sources	Year	LIPUS parameters				Cell	Pathway	Animal model	Disease
			Intensity (mW/cm2)	Frequency (MH)	Time	Total time				
FAK	Xia *et al.* 37	2015	40	3	20min	6 weeks	N/A	integrin/FAK/ MAPK	OA rabbit model	OA
	Jang *et al.* 41	2014	27.5	3.5	5min	N/A	CPC	SFKs/FAK	N/A	PTOA
	Xia *et al.* 19	2019	500/300	1	10/5min	4 days	iPSCs-NCSCs	FAK/ERK1/2	N/A	sciatic nerve injury
	Sato *et al.* 74	2014	30	3	20min	N/A	Rabbit knee synovial membrane cell	integrin/FAk/ MAPK	N/A	RA
	Chen *et al*. 12	2019	30	0.25	20min	2days	BMSCs	FAK/ERK1/2	N/A	femoral defects
	Zhang *et al.* 42	2016	30	1.5	20min	1week	human NP cell	FAK/PI3K/Akt	N/A	IVD degeneration
BDNF	Liu *et al.* 45	2017	110	1	5min	15min	rat astrocyte cell	TrkB-Akt and Calcium-CaMK	N/A	neurodegenerative diseases
	Su *et al.* 78	2017	528	1	5min	3days	N/A	TrkB/AKt-CREB	TBI model	TBI
	Li *et al.* 13	2023	300/80	1	10min	3weeks	Schwann cells	TrkB/Akt/ CREB	bilateral cavernous nerve injury rat	cavernous nerve injury-induced neurogenic erectile dysfunction
TGF-β	Lei *et al.* 101	2015	200/300	1.7	3min	2weeks	N/A	TGF-β1/ Smad/CTGF	ED diabetic rat	diabetic ED
	Yi* et al.* 14	2020	30	1	20min	3/6weeks	N/A	TGF-β1/Smad3	Rabbit TMJOA model	TMJOA
	Ouyang *et al.* 18	2020	60	1	20min	4weeks	N/A	Nrf2/keap1/HO-1 and TGF-β1/Smad	CKD rats	CKD
	Yang *et al.* 87	2014	90	1.5	20min	13days	HPDLC	BMP-Smad	N/A	Fracture healing and osteogenic differentiation
	Bernal *et al.* 9	2015	83	5	15min	N/A	CMAs	BMP-Smad	N/A	ischemic heart disease
HIF	Cao *et al.*^10^	2023	500	1	20min	3days	N/A	ASK1/JNK	MI/R rat	MI/R
	Zhao *et al.* 17	2021	77.2	0.5	20min/ days	5weeks	N/A	HIF-1α/DNMT3a	TAC rat	cardiac fibrosis
	Yang *et al.* 48	2020	45	1	20min	4weeks	Chondrocyte	HIF-1α/HIF-2α	TMJ injury induced by CSD	Temporomandibular disorders
NF-κB	Zhang *et al.* 59	2017	60	1.5	120min	N/A	U937 cells(macrophage)	TLR4-NF-κB	N/A	N/A
	Nagao *et al.* 76	2017	30	1.5	30min	N/A	MC3T3-E1	AT1-PLCβ	N/A	N/A
	Sato *et al.* 53	2015	30	1.5	20min	2weeks	Human salivary gland acinar and ductal cells	upregulates expression of AQP5 and inhibits TNF-α production	N/A	Xerostomia
	Zhao *et al.* 55	2017	200	1.5	20min	3/4times	polyethylene debris induced RAW 264.7 cell	FBXL2-TRAF6	N/A	periprosthetic inflammatory loosening
	Yi *et al.* 56	2021	30	1.5	20min	3days	NP cell	NF-κB	N/A	Intervertebral disc degeneration
CAP	Liu *et al.* 90	2023	N	1	12min	15dys	N/A	N/A	Autoimmune myocarditis mouse	myocarditis
	Zachs *et al.* 75	2019	N	1	20min	N/A	N/A	N/A	OA mouse model	OA
EVs	Liao *et al.* 73	2021	30	1.5	20min	4weeks	N/A	NF-κB	OA rat	OA
	Li *et al.* 61	2023	300	1	15min	N/A	BMSCs	MAPK	N/A	inflammation
	Li *et al.* 62	2018	30	1.5	12h	N/A	BMDCs	TNF-α	N/A	atherosclerosis
	Ye *et al*. 86	2023	80	1	10min	3times	SC	PI3K/Akt/FoxO	BCNI-induced ED rat	NED
Macrophage polarity	Zhang *et al.* 102	2019	30	1.5	20min/ day, 5days/week	2weeks	N/A	N/A	rat model of spinal fusion	inflammation in spinal fusion
	Silva *et al.* 20	2017	40	1	3min	7days	N/A	N/A	cryoinjury of the right TA muscle rat	muscle injuries
	Hsu *et al.* 80	2023	30	1	5min	15min	microglial cell	STAT1/STAT6/ PPARγ	N/A	Neuroinflammation

Abbreviation: FAK: focal adhesion kinase; MAPK: mitogen-activated protein kinase; NF-κB: nuclear factor-κB; OA: osteoarthritis; PTOA: posttraumatic osteoarthritis; CPC: chondrogenic progenitor cell; SFKs: Src family kinases; ERK1/2: extracellular regulated protein kinases 1/2; PI3K: phosphatidylinositol 3-kinase; iPSCs-NCSCs: induced pluripotent stem cells-derived neural crest stem cells; RA: rheumatoid arthritis; NP: nucleus pulposus; IVD: intervertebral disk; BDNF: brain-derived neurotrophic factor; TrkB: tyrosine Kinase receptor B; CaMK: calcium/calmodulin stimulated protein kinase; CREB: cyclic-AMP response binding protein; TBI: traumatic brain injury; TGF-β: transforming growth factor-β; CTGF: connective tissue growth factor; Nrf2/keap1/HO-1: nuclear factor erythroid 2-related factor 2/the Kelch-like ECH-associated protein 1/heme oxygenase 1; ED: erectile dysfunction; HPDLC: human periodontal ligament cell; TMJOA: temporomandibular joint osteoarthritis; CKD: chronic kidney disease; CMAs: Cardiac mesoangioblasts; HIF: hypoxia-inducible factor; ASK1: apoptosis signal-regulating kinase 1; JNK: c-Jun N-terminal kinase; DNMT3a: DNA methyltransferase 3 α; MI/R: myocardial ischemia/reperfusion; TAC: transverse aortic constriction; CSD: chronic sleep deprivation; TLR4: toll like receptor 4; AT1: angiotensin type 1 receptor; PLCβ: phospholipase C β; AQP5: aquaporin Protein-5; FBXL2: F-box and leucine rich repeat protein 2 gene; TRAF6: TNF receptor associated factor 6; FoxO: Forkhead Box O; TMJ: temporomandibular joint; BMSCs: bone marrow-derived mesenchymal stem cells; BMDCs: bone marrow dendritic cells; SC: Schwann cell; CAP: cholinergic anti-inflammatory pathway; BCNI: bilateral cavernous nerve crush injury; NED: neurogenic ED; TA: tibialis anterior; EVs: extracellular vesicles; TNF-α: tumor necrosis factor α; PPARγ: peroxisome proliferator activated receptor γ; STAT1: signal transducer and activator of transcription 1; MC3T3-E1: mouse calvaria osteoblast-like cells; N/A: not applicable.

**Table 2:** Clinical trials published in the last decade on the role of LIPUS in anti-inflammatory and repair

Ref.	Study Type	Study Subjects	Interventions
69	Randomized prospective double-blind placebo-controlled clinical trial	13 patients with a delayed-union of the osteotomized fibula (9 females and 4 males, aged 42 - 63)	LIPUS application: 20 minutes per day for 5 months (intensity: 30mW/cm², burst width: 200μs, frequency: 1.5MHz)
103	Single-blind RCT	150 orthodontic patients (aged 18 - 25)	LIPUS therapy: frequency: 1.6MHz, an average output intensity: 0.2W/cm² for 20 minutes each time (5 minutes for each first molar), with 3 doses
104	Prospective cohort study	21 patients undergoing IVRO surgery (6 males and 15 females, aged 16 - 54)	LIPUS therapy: Started 2 days after surgery, received daily 20-minute LIPUS treatment for 3 weeks (intensity: 30mW/cm², burst width: 200ms, frequency: 1.5MHz)
105	Randomized, open-label, parallel-group, non-inferiority clinical trial	323 patients with mild-to-moderate ED (aged 20 - 60)	Three times per week (3/W) and twice per week: Used a LIPUS treatment with a frequency of 1.7MHz, intensity of 0.3W/cm², a pulse duration time to pulse rest time ratio of 1:4 (200:800ms), 1000Hz for 5 minutes on each side of the penile shaft and crus in turn for a total of 20 minutes per treatment session
68	Randomized controlled clinical trial	40 patients with mandibular fractures (34 males and 6 females, aged 20 - 40 years, ASA II)	Received LIPUS treatment (1.5MHz, 30mW/cm²) on postoperative days 4, 8, 14, and 20 for 20 minutes daily
106	Multicenter, double-blind, parallel RCT	44 patients with Buerger disease and limb ischemia (aged ≥20 years and <65 years, Fontaine III)	Received 20-minute LIPUS treatment once a day for 24 weeks (2.0 MHz pulse signal, 200-second pulse train, 1 kHz repetition rate, intensity of 30 mW/cm², and a duty cycle of 20%)
107	Randomized, double-blind, placebo-controlled trial	106 patients with bilateral KOA (aged ≥40 years, Kellgren & Lawrence II - III)	Received FLIPUS (frequency 0.6MHz, pulse repetition frequency 300Hz, spatial and temporal average intensity 120mW/cm², duty cycle 20%) treatment for 20 minutes a day for 10 days
108	Prospective, randomized, controlled, observer-blinded study	114 patients with knee osteoarthritis (aged ≥40 years, Kellgren - Lawrence I - III)	Received FLIPUS (parameters same as in Document 7) treatment for 20 minutes a day for 12 days
109	Prospective, randomized, double-blind, placebo-controlled study	50 client-owned dogs with naturally occurring unilateral cranial cruciate ligament rupture	Received 20-minute LIPUS treatment daily (1.5MHz, 30mW/cm², 1kHz pulse, 20% duty cycle) for 12 weeks after tibial plateau leveling osteotomy
110	Prospective, randomized, single-blind (assessor), controlled study	40 patients aged 45 - 85 years with painful knee OA	Received TENS treatment or LIPUS combined with TENS treatment
32	RCT	62 patients diagnosed with COVID - 19 pneumonia	Received LIPUS (572 kHz, 50 Hz, 820 mW/cm², 50% duty cycle), twice daily for 30 minutes during hospitalization
111	Prospective, RCT	60 patients with concurrent ED and CP/CPPS	LIPUS treatment or drug therapy with tadalafil and doxazosin or LIPUS combined with tadalafil and doxazosin. Treatment duration was four weeks
100	Systematic review and meta-analysis of RCTs	Seven RCTs with a total of 172 patients undergoing distraction osteogenesis	LIPUS treatment compared with sham devices or no devices
93	Double-blinded, randomized, and placebo-controlled study	25 patients with Buerger disease (12 in the LIPUS group and 13 in the control group)	Received 20-minute LIPUS treatment daily for 24 weeks
112	RCT	45 patients aged 10.5 - 14 years with skeletal class II division 1 malocclusion	Received LIPUS treatment
113	Prospective, randomized, placebo-controlled, single-blind trial	96 patients initially diagnosed with knee osteoarthritis	Intraarticular HADMSCs injection and LIPUS therapy
114	Single blind randomized controlled trial	100 female volunteers with chronic non-specific low back pain	Received Pulsed Laser (3J/cm²) or Pulsed Ultrasound (1W/cm², 3MHz) or Continuous Ultrasound (1W/cm², 1MHz)
115	Single blinded RCT	60 participants with grades II and III KOA	Received HILT and ET or LIPUS and ET or only ET
116	Systematic review and meta-analysis	26 randomized controlled trials with patients having fracture or osteotomy	LIPUS vs sham device or no device
91	Randomized, double-blind, placebo-controlled pilot trial	Patients with refractory angina without indication of PCI or CABG	Received LIPUS treatment or placebo
117	Multicenter double-blind randomized control trial	62 adult patients undergoing limb lengthening or bone transport by distraction osteogenesis	Received active ultrasound device or placebo device
118	RCT	34 patients with early-stage lumbar spondylolysis	Received LIPUS and RPT or only RPT
119	Randomized, blinded, sham controlled clinical trial	501 patients with operatively managed tibial fractures	Received LIPUS treatment
120	Clinical trial	72 patients with TMD (36 with masticatory myositis, 36 with synovitis)	LIPUS treatment for both groups
85	Single-site, prospective, double-blind, RCT	48 participants with clinically confirmed PD	Received LIPUS treatment (600 kHz, 1.0 W/cm2, 50% duty cycle, 30 min/day for 4 months)
121	Meta-analysis	288 participants from 5 RCTs on knee OA	LIPUS treatment vs control

Abbreviation: ASA: American Society of Anesthesiologists; CP/CPPS: chronic prostatitis/chronic pelvic pain syndrome; ED: erectile dysfunction; ET: exercise therapy; FLIPUS: focused low-intensity pulsed ultrasound; GABA: γ-Aminobutyric Acid; IVRO: intraoral vertical ramus osteotomy; HADMSCs: human adipose-derived mesenchymal stem cells; HILT: high intensity laser therapy; KOA: Knee Osteoarthritis; LIPUS: low-intensity pulsed ultrasound; OA: osteoarthritis; PCI: percutaneous coronary intervention; PD: Parkinson disease; RCT: randomized control trial; RPT: routine physiotherapy; TENS: transcutaneous electrical nerve stimulation; TMD: temporomandibular disorders; vs: versus.

**Table 3:** Relationship Between Parameters and Specific Effects

Target Disease/Effect	LIPUS Parameters (Intensity, Frequency, Temporal Mode)	Reference
Bone Repair	30 mW/cm², 1.5 MHz, 20 min/day	64, 66, 68, 109
Osteoarthritis	30-45 mW/cm², 1-1.5 MHz, 20 min/day	16, 37, 72, 74, 107
Nerve Repair	80-300 mW/cm², 1 MHz, 10-20 min/day	13, 45, 78, 80
Cardiovascular Disease	77.2-500 mW/cm², 0.5-1 MHz, 20 min/day	10, 17
Chronic Inflammation (e.g., Prostatitis)	Parameters not explicitly detailed, but clinical efficacy shown	15, 26
Extracellular Vesicle Regulation	300 mW/cm², 1-1.5 MHz, 12-15 min	61, 62
Muscle Injury	40-250 mW/cm², 1 MHz, 3-20 min/day	58, 95, 96
Intervertebral disc degeneration	30 mW/cm², 1.5 MHz, 20 min/day	42, 56

## Abbreviations

ADSC-Exos: adipose-derived stem cell exosomes
AT1: angiotensin type 1 receptor
ASK1: apoptosis signal-regulating kinase 1
AQP5: aquaporin Protein-5
BCNI: bilateral cavernous nerve crush injury
BDNF: brain-derived neurotrophic factor
BMSCs: bone marrow-derived mesenchymal stem cells
BMDCs: bone marrow dendritic cells
SC: schwann cell
BMMs: bone marrow mononuclear cells
CAP: cholinergic anti-inflammatory pathway
CaMK: calcium/calmodulin stimulated protein kinase
CREB: cyclic-AMP response binding protein
CP/CPPS: chronic prostatitis/chronic pelvic pain syndrome
CPC: chondrogenic progenitor cell
SFKs: Src family kinases
CTGF: connective tissue growth factor
CKD: chronic kidney disease
CMAs: Cardiac mesoangioblasts
DNMT3a: DNA methyltransferase 3 α
ED: erectile dysfunction
ER: endoplasmic reticulum
ERK1/2: extracellular regulated protein kinases 1/2
EVs: extracellular vesicles
FAK: focal adhesion kinase
FBXL2: F-box and leucine rich repeat protein 2 gene
FoxO: Forkhead Box O
HPDLC: human periodontal ligament cell
iPSCs-NCSCs: induced pluripotent stem cells-derived neural crest stem cells
HIF: hypoxia-inducible factor
HUVECs: human umbilical vein endothelial cells
IVD: intervertebral disk
JNK: c-Jun N-terminal kinase
LIPUS: low intensity pulse ultrasound
MAPK: mitogen-activated protein kinase
LTCCs: L-type calcium channels
MI/R: myocardial ischemia/reperfusion
NF-κB: nuclear factor-κB
OA: osteoarthritis
NP: nucleus pulposus
Nrf2/keap1/HO-1: nuclear factor erythroid 2-related factor 2/the Kelch-like ECH-associated protein 1/heme oxygenase 1
NED: neurogenic ED
PTOA: posttraumatic osteoarthritis
PI3K: phosphatidylinositol 3-kinase
PLCβ: phospholipase C β
PPARγ: peroxisome proliferator activated receptor γ
PDLSCs: periodontal ligament stem cells
RA: rheumatoid arthritis
RIPK1: receptor-interacting protein kinase 1
STAT1: signal transducer and activa tor of transcription 1
SCs: Schwann cells
TA: tibialis anterior
TrkB: tyrosine Kinase receptor B
TBI: traumatic brain injury
TGF-β: transforming growth factor-β
TMJOA: temporomandibular joint osteoarthritis
TLR4: toll like receptor 4
TAC: transverse aortic constriction
TRAF6: TNF receptor associated factor 6
TMJ: temporomandibular joint
TNF-α: tumor necrosis factor α
YAP: yes associated protein
